# Multi-Agent Deep Reinforcement Learning for Joint Task Offloading and Resource Allocation in IIoT with Dynamic Priorities

**DOI:** 10.3390/s25196160

**Published:** 2025-10-04

**Authors:** Yongze Ma, Yanqing Zhao, Yi Hu, Xingyu He, Sifang Feng

**Affiliations:** 1 Shenyang Institute of Computing Technology, Chinese Academy of Sciences, Shenyang 110168, China; mayongze20@mails.ucas.ac.cn (Y.M.); zhaoyanqing20@mails.ucas.ac.cn (Y.Z.);; 2 University of Chinese Academy of Sciences, Beijing 101408, China

**Keywords:** Industrial Internet of Things, task offloading, resource allocation, multi-agent deep reinforcement learning, cloud–edge–end collaboration

## Abstract

**Highlights:**

**What are the main findings?**

- Developed a cloud–edge–end collaborative framework that jointly optimizes task offloading and resource allocation for IIoT systems with dynamic task priorities.
- Designed a priority-gated attention-enhanced MAPPO algorithm to capture priority-related features and improve decision accuracy under fluctuating workloads.

**What is the implication of the main finding?**

- Improves system adaptability and efficiency in IIoT environments with fluctuating workloads and heterogeneous QoS demands.
- Enables robust, low-latency, and energy-efficient scheduling for cloud–edge–end collaborative systems.

**Abstract:**

The rapid growth of Industrial Internet of Things (IIoT) terminals has resulted in tasks exhibiting increased concurrency, heterogeneous resource demands, and dynamic priorities, significantly increasing the complexity of task scheduling in edge computing. Cloud–edge–end collaborative computing leverages cross-layer task offloading to alleviate edge node resource contention and improve task scheduling efficiency. However, existing methods generally neglect the joint optimization of task offloading, resource allocation, and priority adaptation, making it difficult to balance task execution and resource utilization under resource-constrained and competitive conditions. To address this, this paper proposes a two-stage dynamic-priority-aware joint task offloading and resource allocation method (DPTORA). In the first stage, an improved Multi-Agent Proximal Policy Optimization (MAPPO) algorithm integrated with a Priority-Gated Attention Module (PGAM) enhances the robustness and accuracy of offloading strategies under dynamic priorities; in the second stage, the resource allocation problem is formulated as a single-objective convex optimization task and solved globally using the Lagrangian dual method. Simulation results show that DPTORA significantly outperforms existing multi-agent reinforcement learning baselines in terms of task latency, energy consumption, and the task completion rate.

## 1. Introduction

The Industrial Internet of Things (IIoT) is a core application of the Internet of Things (IoT) in industrial domains. By interconnecting distributed industrial equipment, edge nodes, and cloud platforms, IIoT enables real-time sensing, intelligent decision-making, and efficient execution throughout production processes, thereby substantially improving the flexibility and intelligence of industrial systems [1]. Driven by rapid adoption, IIoT devices are being deployed across manufacturing, transportation, and energy, among other critical sectors; by the end of 2025, the global number of IoT devices is projected to reach 75.44 billion [2]. However, this proliferation generates compute-intensive and heterogeneous data-processing workloads that exceed the capabilities of local devices. Consequently, effective task scheduling and resource allocation have become central research problems in IIoT [3].

To address these challenges, cloud and edge computing have emerged as complementary paradigms. Cloud computing aggregates massive resources and leverages virtualization for elastic provisioning [4], which is well suited to large-scale analytics; yet, as the number of connected devices surges, bandwidth bottlenecks, and end-to-cloud transmission latency hinder real-time responsiveness. Edge computing pushes computation toward data sources to reduce backhaul and response latency [5,6], but constrained compute, memory, and energy budgets at edge/endpoint devices limit their ability to handle complex workloads independently. To exploit the strengths of both, cloud–edge–end (CEE) collaboration has gained prominence as a preferred computing paradigm [7,8], denoting a layered system in which cloud data centers, edge servers, and end devices cooperate. Within this architecture, tasks are dispatched across cloud, edge, and end according to computational demand, latency constraints, and system state, thereby improving overall resource utilization [9]. Given that multiple tasks contend for limited compute and bandwidth, joint execution must be accompanied by optimized resource allocation to satisfy IIoT requirements for Quality of Experience (QoE) and Quality of Service (QoS). Because greater computational provisioning typically incurs higher power consumption, task offloading (TO) and resource allocation (RA) have become key techniques for alleviating compute bottlenecks, balancing load, reducing latency, and improving system efficiency [10].

Traditional TO-RA methods are usually based on static priority assignment and rely on predefined rules to allocate tasks to different computing nodes [11]. These methods can handle some common fixed workloads. However, in industrial scenarios, task priorities and resource demands may change rapidly due to dynamic production environments [12,13]. Relying solely on static priority for task scheduling cannot effectively adapt to such changes, leading to low system resource utilization and increased task response delays. Some existing studies have proposed using deep learning and reinforcement learning techniques for TO-RA of priority tasks [14,15,16]. However, in real IIoT environments, multitasking, heterogeneous resources, and dynamic conditions affect the computational efficiency of traditional iterative methods, deep learning, and reinforcement learning approaches, resulting in reduced decision-making timeliness and accuracy [17]. Moreover, existing centralized decision-making methods suffer from significantly increased computational complexity and communication overhead in distributed environments with dynamically changing task priorities. Therefore, multi-agent deep reinforcement learning (MADRL) methods based on the centralized training and decentralized execution (CTDE) framework, such as Multi-Agent Deep Deterministic Policy Gradient (MADDPG) and Multi-Agent Proximal Policy Optimization (MAPPO), have become mainstream paradigms for solving complex distributed task scheduling problems [18,19,20]. These methods leverage global state and action information during training to improve policy stability and use local observations during execution for independent decision-making. This approach balances cooperative optimization ability and practical deployment scalability, enabling efficient TO-RA in distributed environments [21,22].

This study addresses the task offloading and resource allocation problem for dynamic-priority tasks in a cloud–edge–end collaborative environment. We first design a cloud–edge–end collaborative architecture capable of perceiving dynamic task priorities. Then, we propose a novel two-stage task offloading and resource allocation approach, named DPTORA. By integrating MADRL with convex optimization techniques, DPTORA enables efficient task scheduling and resource management in dynamically changing industrial environments, improving task offloading accuracy while reducing system latency and energy consumption. The main contributions of this work are summarized as follows:

1. We construct a cloud–edge–end collaborative framework for TO-RA, and we design a dynamic multi-level feedback queue task-scheduling model. In this framework, the optimization objective for collaborative TO-RA is to minimize overall system latency and energy costs. The problem is formulated as a Mixed-Integer Nonlinear Programming (MINLP) problem. To address this, we propose a DPTORA algorithm, which provides efficient solutions for dynamically prioritized industrial tasks.
2. In the first stage, we formulate the task offloading subproblem as a Partially Observable Markov Decision Process (POMDP). To address this, we propose a MAPPO algorithm enhanced with a Priority-Gated Attention Mechanism (PGAM). By incorporating PGAM into the policy network, agents are able to adaptively focus on task priority features, thereby improving the sensitivity of decision-making and the accuracy of resource allocation under dynamic priority conditions.
3. In the second stage, we formulate the RA subproblem for each edge server as a constrained, weighted single-objective convex optimization problem. By applying the Karush–Kuhn–Tucker (KKT) conditions, we analytically explore the duality of the objective function. Through the construction of a Lagrange multiplier function, we decouple the transmission and computation resource constraints, and ultimately derive closed-form globally optimal allocation strategies for both bandwidth and computational resources.
4. We conduct extensive simulation experiments to validate the convergence and effectiveness of the proposed DPTORA algorithm. The results demonstrate that DPTORA outperforms baseline methods and other mainstream MADRL methods in terms of the task response time, system energy consumption, and the task completion rate.

The remainder of this paper is organized as follows. Section 2 reviews related work. Section 3 formulates the system model and optimization objectives. Section 4 details the proposed DPTORA algorithm. Section 5 presents the experimental results and analysis. Section 6 concludes the paper.

## 2. Related Works

In the IIoT environment, the primary objectives of TO-RA technologies include reducing computational latency, optimizing resource utilization, and improving system energy efficiency [3,8]. However, traditional TO-RA strategies, which are mostly based on static rules, struggle to adapt to the highly heterogeneous, dynamic nature, and stringent real-time requirements of IIoT systems. Consequently, in recent years, researchers have proposed various intelligent offloading strategies, including game-theoretic approaches, deep learning optimization, and reinforcement learning-based offloading, to elevate the intelligence level of offloading decisions.

### 2.1. Traditional TO-RA Approaches

Early TO-RA approaches primarily relied on centralized heuristic algorithms, mathematical optimization models, and static rules. Heuristic algorithms aim to quickly find approximate optimal solutions based on empirical rules or heuristic strategies. Tran et al. [23] proposed a joint TO-RA optimization method using heuristic algorithms to reduce task completion time and energy consumption. However, heuristic algorithms may fall into local optima during the solution search process and typically exhibit poor decision-making timeliness in dynamic environments. Game theory has been widely applied in scenarios involving collaborative task offloading (TO) among multiple devices. Cooperative games facilitate collaboration among multiple devices by enabling task sharing and resource scheduling to optimize global system performance. In [24], a multi-hop cooperative computation offloading method was proposed for Industrial Internet of Things (IIoT) environments. It employs game theory to optimize TO decisions, enabling devices to share resources and coordinate tasks and thereby minimizing computation time and energy consumption while improving task processing efficiency. Similarly, Ding et al. [25] aimed to minimize energy consumption by leveraging game-theoretic approaches to optimize task assignment and resource utilization in large-scale IoT systems. On the other hand, non-cooperative game models allow IIoT devices to autonomously decide whether to offload tasks without sharing private information, thereby maximizing their individual utility. Teng et al. [26] proposed a TO method based on non-cooperative game theory in a mobile edge computing (MEC) scenario, which achieves profit maximization while balancing task completion time and energy consumption.

### 2.2. Machine Learning-Based TO-RA Approaches

Although game-theoretic approaches can effectively coordinate offloading decisions among multiple devices, the vast number of devices in IIoT networks leads to an enormous strategy space, making it difficult for such algorithms to converge in real time. As a result, an increasing number of researchers are turning to machine learning (ML) and reinforcement learning (RL) methods to optimize TO-RA strategies. Traditional ML methods, such as Support Vector Machines (SVMs) and Deep Neural Networks (DNNs), can predict optimal offloading strategies based on historical data [27]. However, these methods typically require large volumes of training data and exhibit weak generalization capabilities, making them poorly suited to dynamic IIoT environments. In contrast, Deep Reinforcement Learning (DRL) enables IIoT devices to continuously learn optimal offloading policies through interaction with the environment. Deng et al. [28] proposed an intelligent delay-aware partial TO system for multi-user IIoT scenarios, which uses Q-learning and Deep Deterministic Policy Gradient (DDPG) to optimize offloading decisions, significantly reducing task processing delay and improving service quality. Chai et al. [29] addressed the problem of multi-task offloading and resource allocation in multi-task mobile edge computing systems, and they proposed an attention mechanism combined with Proximal Policy Optimization (PPO) to compute offloading strategies. While DRL methods have demonstrated strong performance in task offloading and resource scheduling, their single-agent decision-making frameworks face significant challenges in IIoT systems with multi-node collaboration and complex dynamic environments. These limitations include low training efficiency, isolated decision-making, and poor adaptability to multi-task cooperative scenarios.

### 2.3. Multi-Agent Deep Reinforcement Learning Approaches

In recent years, MADRL has emerged as a research hotspot in TO-RA for IIoT systems, owing to its superior collaborative decision-making capabilities. Du et al. [30] proposed a MADDPG-based TO strategy in a comprehensive air-ground integrated MEC framework, enabling efficient cooperation among multiple edge nodes. Their method achieved significant energy savings while ensuring service timeliness. Suzuki et al. [31] designed a collaborative MADRL-based scheduling framework tailored for multi-cloud and multi-edge networks. By incorporating value function decomposition, they achieved a coordinated trade-off between local autonomy and global optimization, improving the utilization of server and link resources and reducing task delay. To address communication bottlenecks in multi-agent systems, Yao et al. [32] introduced a GAT-based MADRL framework. Using graph attention networks (GATs), the method dynamically focused on critical state information, thereby reducing communication overhead and enhancing decision efficiency.

Despite these advancements, only a limited number of studies have addressed the differentiated requirements of heterogeneous industrial tasks with varying priorities [31,33,34]. For instance, Xu et al. [33] developed a D3QN-based priority-aware offloading scheme that incorporated a deadline-driven reward mechanism to meet real-time constraints under varying interference conditions. Uddin et al. [35] assigned static priorities to vehicular edge computing tasks and introduced a prioritized deep Q-network (DQNP), where each priority level received a tailored reward structure. This improved the completion rate of high-priority tasks while reducing overall energy consumption, delay, and resource scarcity. Dai et al. [34] proposed a DDPG-based TO-RA scheme that regulated network access for tasks with different priorities, maximizing overall system utility in a priority-aware manner. However, most of these methods adopt static priority settings, which fail to capture the dynamic and context-sensitive nature of industrial task priorities in real-world IIoT environments.

Table 1 summarizes the contributions of recent studies. Our work jointly considers latency, energy consumption, and dynamic priorities while accounting for the complexity induced via multi-tasking, heterogeneous resources, and time-varying conditions in cloud–edge–end collaborative environments. We propose a two-stage framework for task offloading and resource allocation. The framework employs a dynamic multi-level feedback queue to adaptively update task priorities and couples multi-agent deep reinforcement learning with a resource-allocation module for joint optimization, thereby yielding near-optimal task placement and scheduling in cloud–edge–end settings and offering a practical solution to improve end-to-end IIoT performance.

## 3. System Model

This section presents the proposed cloud–edge–end collaborative architecture, which integrates the high computational power of cloud computing, the low-latency benefits of edge computing, and a dynamic priority-aware mechanism to enable an efficient TO-RA strategy. The architecture encompasses a network model, a task model, a communication model, a computation model, and a dynamic priority queue model. The mathematical notations used throughout this paper are summarized in Table 2.

### 3.1. Network Model

**Figure 1:**
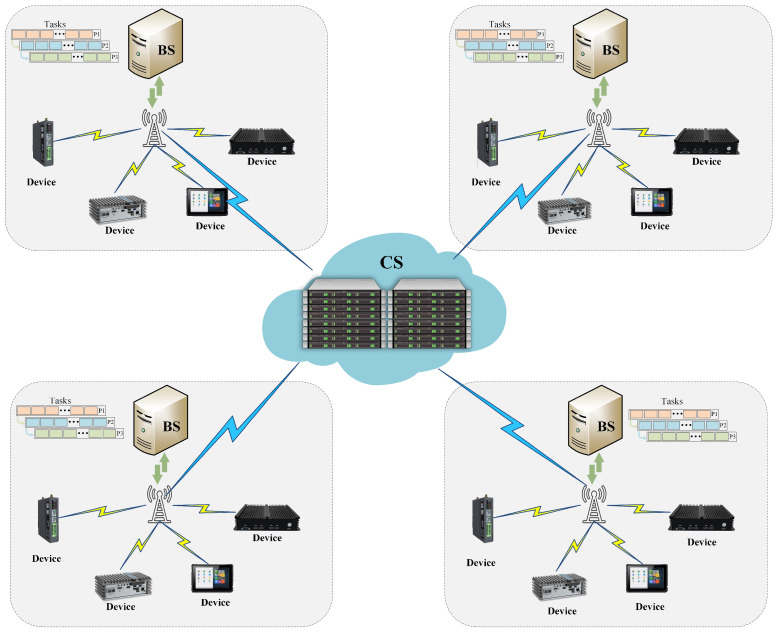
Diagram of the cloud–edge–end collaborative network architecture.

Figure 1 shows the proposed cloud–edge–end collaborative architecture designed for IIoT applications. This architecture comprises three distinct components: IIoT devices, edge servers (ESs), and a cloud server cluster (CS) [9]. The IIoT devices include a wide range of intelligent terminals such as sensors, controllers, industrial robots, and smart machine tools, which are distributed across industrial sites. These devices are collectively denoted as the set $D=\{D_{1},D_{2},\ldots ,D_{N}\}.$ They continuously generate tasks while establishing connections with the nearest ES through the low-power, lightweight Message Queuing Telemetry Transport (MQTT) protocol. [36]. The system includes multiple edge servers, represented as the set $E=\{E_{1},E_{2},\ldots ,E_{M}\}$, and a cloud computing cluster composed of multiple cloud servers. Each edge server, $E_{m}$, is associated with a subset of IIoT devices, $U_{m}\subseteq D$, where $\left|U_{m}\right|$ denotes the number of IIoT devices connected to $E_{m}$. In accordance with practical industrial deployments, each edge server communicates with other ES via a local area network (LAN) and connects to the CS via fiber-optic links or high-speed wireless Internet [37].

### 3.2. Task Model

Following the approach in [38], we divide the entire timeline into *L* equal-length time slots, denoted as $T=\{t_{1},t_{2},\ldots ,t_{L}\}$. In each time slot, t∈T, it is assumed that an IIoT terminal device, $D_{n}$, randomly generates an industrial task, $R_{n}^{t}$, defined as $R_{n}^{t}=\left\{d_{n}^{t},c_{n}^{t},\phi_{n}^{t},P_{n}^{t,ini}\right\}$, where $d_{n}^{t}$ represents the input data size required for task execution, $c_{n}^{t}$ denotes the number of CPU cycles needed to complete the task, $\phi_{n}^{t}$ is the deadline of task $R_{n}^{t}$, and $P_{n}^{t,ini}$ denotes its initial priority level. The task $R_{n}^{t}$ can be executed locally on the device, offloaded to an ES, or further offloaded to the CS. A task is considered failed if it is not completed before its deadline, $\phi_{n}^{t}$.

In line with previous work [15], each industrial task can be decomposed into multiple subtasks that are executed in parallel at different computing nodes to maximize the utilization of system resources. As shown in Figure 1, each industrial task can be processed in parallel across IIoT devices, ES, and the CS, thereby fully utilizing available system resources.

We define the offloading ratio decision set as $x=\{x_{n,n}^{t},x_{n,m}^{t}\},\;\forall n\in D,\;m\in E$, where $x_{n,n}^{t}\in \left[0,1\right]$ indicates the proportion of the task processed locally on device $D_{n}$, $\left(1-x_{n,n}^{t}\right)\cdot x_{n,m}^{t}\in \left[0,1\right]$ represents the proportion of the task offloaded to edge server $E_{m}$, and $\left(1-x_{n,n}^{t}\right)\cdot \left(1-x_{n,m}^{t}\right)\in \left[0,1\right]$ denotes the proportion of the task offloaded to the CS. This offloading mechanism enables flexible task allocation and parallel execution, effectively improving system responsiveness and resource utilization efficiency.

### 3.3. Communication Model

The communication model characterizes the transmission process of task data among IIoT devices, ES, and the CS within an IIoT system. To ensure high efficiency and reliability, differentiated communication technologies are adopted across different transmission links: Orthogonal Frequency Division Multiple Access (OFDMA) is employed between IIoT devices and ES to mitigate interference, while fixed-bandwidth wired or high-speed wireless connections are used for communication between ES and the CS. The two types of communication links are described in detail below.

The communication link between IIoT devices and ES is one of the most critical bottlenecks in the system, as its quality directly impacts the real-time responsiveness of task execution. Given the large number of devices and limited spectral resources, OFDMA is employed to support efficient multi-device access and interference management. Compared with traditional multiple-access schemes such as Frequency Division Multiple Access (FDMA) or Time Division Multiple Access (TDMA), OFDMA divides the available bandwidth into multiple mutually orthogonal subcarriers. Each device occupies one or more subcarriers for parallel data transmission, significantly reducing inter-device interference and improving spectral efficiency [39,40]. This feature is particularly crucial for satisfying the high concurrency and low latency requirements of industrial scenarios. In OFDMA communication, transmission quality is determined by the signal-to-noise ratio (SNR) of the channel.

Assume that, during time slot *t*, the channel gain between IIoT device $D_{n}$ and edge server $E_{m}$ is $h_{n,m}^{t}$, the transmission power is $P_{n,m}^{t}$, the noise power spectral density is $N_{0}$, and the allocated bandwidth is $B_{n,m}^{t}$. Then, the instantaneous SNR can be expressed as

(1) $${\mathrm{SNR}}_{n,m}^{t}=\frac{P_{n,m}^{t}\cdot {\left|h_{n,m}^{t}\right|}^{2}}{N_{0}\cdot B_{n,m}^{t}}$$

Based on Shannon’s formula, the theoretical data transmission rate from device $D_{n}$ to edge server $E_{m}$ is given by

(2) $$r_{n,m}^{t}=B_{n,m}^{t}\cdot \log_{2}\left(1+{\mathrm{SNR}}_{n,m}^{t}\right)$$

this rate determines the device’s ability to upload task data to the ES.

In contrast to device-side communication, the link between ES and the CS typically utilizes high-speed and stable transmission channels such as fiber broadband or high-speed Ethernet. To simplify modeling in industrial scenarios, it is assumed that the transmission rate between each ES and the CS is constant, denoted as $R_{E,CS}$ (in MB/s). This rate is used to quantify the transmission overhead when a task is further offloaded from the edge to the cloud and directly affects the overall task completion latency and energy consumption. However, in practical deployments, the backhaul link between edge servers and the cloud can be affected by factors such as network congestion and bandwidth constraints, leading to fluctuations in the transmission rate. Accordingly, while this assumption simplifies the model analysis, it also introduces limitations; future work can incorporate dynamic network conditions to refine the system design and better reflect real-world scenarios.

### 3.4. Computational Model

To evaluate the Quality of Service (QoS) in IIoT systems, this paper considers task execution delay and energy consumption as key performance metrics. Task delay comprises two main components: computation delay and data transmission delay. Energy consumption refers to the electrical energy used by devices or servers during task processing.

In the proposed cloud–edge–end collaborative architecture, industrial tasks generated via IIoT devices can be processed through local execution or offloaded execution, where both modes can operate in parallel to enhance system efficiency. Therefore, this study focuses on modeling and analyzing three performance indicators—transmission delay, computation delay, and energy consumption—under different computation scenarios: local, edge, and cloud. These models provide a theoretical foundation for the subsequent TO-RA strategies.

**(1) Local Computation**

The local computation model focuses on the delay and energy consumption when tasks are processed on the IIoT device itself. At time slot *t*, the local computation delay $T_{\mathrm{local},n}^{t}$ and energy consumption $E_{\mathrm{local},n}^{t}$ for device $D_{n}$ are given as follows:

(3) $$T_{\mathrm{local},n}^{t}=x_{n,n}^{t}\cdot \frac{c_{n}^{t}}{f_{n}^{t}}$$

(4) $$E_{\mathrm{local},n}^{t}=\kappa_{n}\cdot {f_{n}^{t}}^{2}\cdot c_{n}^{t}\cdot x_{n,n}^{t}$$

where $f_{n}$ is the computing capability of the device, and $\kappa_{n}$ is a hardware-specific constant that reflects the relationship between energy consumption and operating frequency.

**(2) Offloaded Computation**

Given the limited computing capacity of IIoT devices, part of the task can be offloaded to a connected edge server, $E_{m}$, or a remote CS for processing. We model these two offloading scenarios as follows:

*a. Edge Offloading*

When a task is offloaded to an ES, the uplink transmission delay and computation delay are computed as follows:

(5) $$T_{\mathrm{tran},n,m}^{t}=\left(1-x_{n,n}^{t}\right)\cdot x_{n,m}^{t}\cdot \frac{d_{n}^{t}}{r_{n,m}^{t}}$$

(6) $$T_{\mathrm{comp},n,m}^{t}=\left(1-x_{n,n}^{t}\right)\cdot x_{n,m}^{t}\cdot \frac{c_{n}^{t}}{f_{m,n}^{t}}$$

where $d_{n}^{t}$ is the task data size, $r_{n,m}^{t}$ is the uplink transmission rate from device $D_{n}$ to edge server $E_{m}$, and $f_{m,n}^{t}$ denotes the computing resources allocated via the ES to device $D_{n}$.

Thus, the total delay of offloading to the ES is as follows:

(7) $$T_{\mathrm{Edge},n,m}^{t}=T_{\mathrm{tran},n,m}^{t}+T_{\mathrm{comp},n,m}^{t}$$

The corresponding energy consumption due to wireless transmission is as follows:

(8) $$E_{\mathrm{tran},n,m}^{t}=P_{\mathrm{tran},n,m}\cdot T_{\mathrm{tran},n,m}^{t}$$

where $P_{\mathrm{tran},n,m}$ is the transmission power of device $D_{n}$. The total energy consumption of device $D_{n}$ is then given by the sum of local computation energy and offloading transmission energy:

(9) $$E_{n}^{t}=E_{\mathrm{local},n}^{t}+E_{\mathrm{tran},n,m}^{t}$$

*b. Cloud Offloading*

When a task is further offloaded to the cloud, the total delay includes three components: transmission from the device to the ES, transmission from the ES to the CS, and cloud-side computation. The total delay for cloud offloading is given as follows:

(10) $$T_{\mathrm{Cloud},n,m}^{t}=T_{\mathrm{tran},n,m}^{t}+\left(1-x_{n,n}^{t}\right)\left(1-x_{n,m}^{t}\right)\cdot \left(\frac{d_{n}^{t}}{R_{E,CS}}+\frac{c_{n}^{t}}{f_{cs}}\right)$$

where $R_{E,CS}$ is the transmission rate from the ES to the CS, and $f_{cs}$ is the computing capability of the CS.

**(3) Overall Task Completion Delay**

As tasks can be executed in parallel across the local device, ES, and CS, the total task completion time is determined by the maximum delay among the three computation modes:

(11) $$T_{n}^{t}=\max\left(T_{\mathrm{local},n}^{t},T_{\mathrm{Edge},n,m}^{t},T_{\mathrm{Cloud},n,m}^{t}\right)$$

### 3.5. Dynamic Priority Queue Model

In the IIoT environment, tasks exhibit characteristics such as high dynamism, strong real-time requirements, and heterogeneity, which place higher demands on task scheduling at the edge layer. To address this, this paper proposes a dynamic priority scheduling mechanism based on the M/M/1 queuing model [41,42]. This mechanism enhances overall system scheduling efficiency and resource utilization by establishing an initial priority assignment driven by task characteristics, a runtime state-aware dynamic priority adjustment mechanism, and a multi-level priority scheduling queue incorporating system load feedback.

#### 3.5.1. Initial Priority Assignment

When a task enters the system, its initial priority is assigned based on its inherent characteristics. The specific formula is as follows:

(12) $$P_{i}^{t,ini}=\alpha \cdot \frac{d_{\max}-d_{i}^{t}}{d_{\max}-d_{\min}}+\beta \cdot \frac{c_{\max}-c_{i}^{t}}{C_{\max}-C_{\min}}+\gamma \cdot \frac{\phi_{\max}-\phi_{i}^{t}}{\phi_{\max}-\phi_{\min}}$$

where $d_{i}^{t}$ is the data size of the task, $c_{i}^{t}$ is the computational requirement, $\phi_{i}^{t}$ is the latest completion time of the task, $\left(d_{\max},d_{\min},c_{\max},c_{\min},\phi_{\max},\phi_{\min}\right)$ denote the maximum and minimum values of these characteristics among historical tasks, and α, β, and γ are weight coefficients that satisfy α+β+γ=1, which reflect the influence of different task characteristics on the priority level and can be adjusted based on specific scenarios. This formula normalizes the task features to the [0,1] interval. The initial priority $P_{i}^{t,\mathrm{ini}}$ thus falls within the range [0,3], where a higher value indicates a more urgent task requiring prioritized scheduling.

#### 3.5.2. Dynamic Priority Adjustment

In IIoT environments, task states change over time, rendering static priority mechanisms inadequate for accurately reflecting the real-time urgency of tasks. To address this limitation, we propose a dynamic priority adjustment mechanism that integrates task waiting time, system load, and task urgency.

The task waiting time refers to the duration a task spends in the queue after entering the system. As this waiting time increases, the task’s priority should be correspondingly elevated to prevent it from being indefinitely delayed. A dynamic adjustment factor is introduced to linearly increase the priority based on waiting time, defined as follows:

(13) $$p_{i}^{t,wait}=\delta \cdot \frac{w_{i}^{t}}{T_{ts}}$$

where $w_{it}$ denotes the current waiting time of task *i*, and $T_{ts}$ is a predefined threshold representing the maximum acceptable waiting time. Once this threshold is exceeded, the task’s priority increases significantly. The coefficient δ regulates the degree to which waiting time impacts the priority. This mechanism allows the system to dynamically respond to waiting conditions and allocate more computational resources to long-waiting tasks without disrupting the scheduling of high-priority tasks.

The system load reflects the current utilization level of computing resources and is typically normalized within the range [0,1]. Under high-load conditions, task scheduling must be handled with greater caution to prevent system overload. To regulate the rationality of priority escalation, we define a load-based adjustment factor as follows:

(14) $$p_{i}^{t,load}=1-\eta \cdot L$$

where L∈[0,1] represents the current load of the ES, and η is a tuning coefficient controlling how strongly system load suppresses priority escalation. This mechanism mitigates unregulated priority inflation during high-load periods, ensuring stable scheduling and fair resource allocation.

Task urgency quantifies the time sensitivity of a task, calculated based on the remaining time until its latest completion deadline. Tasks with higher urgency are prioritized. The urgency adjustment factor is given by:

(15) $$p_{i}^{t,urgency}=\frac{\phi_{i}^{t}-T_{\mathrm{current}}}{\phi_{i}^{t}-T_{\mathrm{arrival}}}$$

where $\phi_{it}$ is the task’s latest allowable completion time, $T_{\mathrm{current}}$ is the current system time, and $T_{\mathrm{arrival}}$ is the task’s arrival time. This formulation ensures that tasks nearing their deadlines receive higher scheduling priority, thereby balancing latency constraints with resource distribution across tasks of varying urgency.

By integrating the above three factors, the final dynamic priority of a task is computed as follows:

(16) $$P_{i}^{t,dyn}=P_{i}^{t,ini}+p_{i}^{t,wait}\cdot p_{i}^{t,load}\cdot p_{i}^{t,urgency}$$

This dynamic priority model comprehensively considers task characteristics, system state, and time urgency to adaptively adjust scheduling priorities, thereby enhancing the system’s responsiveness and scheduling adaptability.

#### 3.5.3. Multi-Level Priority Feedback Queue Scheduling

During the scheduling execution phase, to improve task scheduling efficiency and resource utilization, the conventional single-queue structure is extended into a multi-level priority queue structure. Each ES node maintains two internal queues: a high-priority queue, $Q_{1}$, and a low-priority queue, $Q_{2}$. The high-priority queue, $Q_{1}$, stores tasks with a relatively high dynamic priority, typically those with stringent timeliness requirements. To minimize latency, these tasks are preferentially processed and offloaded via the ES. In contrast, the low-priority queue $Q_{2}$ contains tasks with lower priority or low sensitivity to delay. These tasks are directly offloaded to the CS, $\left\{x_{n,n}^{t}=0,x_{n,m}^{t}=0\right\}$, thereby fully leveraging the cloud’s abundant computational resources while reducing competition for local edge and device resources. This design protects the scheduling order and processing efficiency of high-priority tasks. The system incorporates a periodic feedback mechanism to monitor queue status and scheduling effectiveness in real time, dynamically reassessing task priorities and flexibly allocating tasks to appropriate queues. This enables differentiated resource allocation and tailored processing strategies.

Task queue assignment is governed by a dynamically adjusted priority threshold, denoted as $P_{\mathrm{threshold}}^{t}$, which combines task urgency and current system load to meet diverse scheduling demands. At each time slot, *t*, the system calculates the dynamic priority, $p_{k}^{t,\mathrm{dym}}$, for each task and considers the current system load, *L*, to determine queue placement. The threshold is computed using the following formula:

(17) $$P_{\mathrm{threshold}}^{t}=\mu \cdot \frac{1}{\left|Q\right|}\sum_{k=1}^{\left|Q\right|}p_{k}^{t,dym}+\nu \cdot L$$

where μ and ν are tuning coefficients that balance the influence of the average priority and the system load. The term $\frac{1}{\left|Q\right|}\sum_{k=1}^{\left|Q\right|}p_{k}^{t,dym}$ represents the average dynamic priority of the current task set *Q* on the ES, reflecting the global priority distribution. This threshold is not static; it adjusts adaptively based on real-time system conditions.

At each time slot, the system periodically re-evaluates task priorities and reallocates tasks to the appropriate queues based on $P_{\mathrm{threshold}}^{t}$. Tasks exceeding the threshold are directed to the high-priority queue, while others are relegated to the low-priority queue. The detailed scheduling procedure is outlined in Algorithm 1.
**Algorithm 1** Multi-level priority feedback queue scheduling
  1: **Input:** Task set $T=\{T_{1},T_{2},\ldots ,T_{n}\}$
  2: **Output:** Task queue allocation $Q_{1}$, $Q_{2}$ at each ES
  3: **while** system is running **do**
  4:      **for** each $T_{i}\in T$ **do**
  5:             Compute initial priority $P_{i}^{t,\mathrm{ini}}$
  6:             Compute waiting time factor $p_{i}^{t,\mathrm{wait}}$
  7:             Compute load suppression factor $p_{i}^{t,\mathrm{load}}$
  8:             Compute urgency factor $p_{i}^{t,\mathrm{urgency}}$
  9:             Calculate dynamic priority:             $P_{i}^{t,\mathrm{dyn}}=P_{i}^{t,\mathrm{ini}}+p_{i}^{t,\mathrm{wait}}\cdot p_{i}^{t,\mathrm{load}}\cdot p_{i}^{t,\mathrm{urgency}}$
10:      **end for**
11:      Calculate dynamic priority threshold $P_{\mathrm{threshold}}^{t}$ using Equation (17)
12:      **for** each $T_{i}\in T$ **do**
13:             **if** $P_{i}^{t,\mathrm{dyn}}>P_{\mathrm{threshold}}^{t}$ **then**
14:                  Assign $T_{i}$ to high-priority queue $Q_{1}$
15:             **else**
16:                  Assign $T_{i}$ to low-priority queue $Q_{2}$
17:             **end if**
18:      **end for**
19:      Execute tasks in $Q_{1}$ using offloading and resource allocation policies
20:      Offload tasks in $Q_{2}$ to the CS
21: **end while**


### 3.6. Problem Formulation

In the cloud–edge–end collaborative computing architecture proposed in this paper, IIoT terminal devices can selectively offload parts of their tasks to ES or the CS based on task characteristics, thereby alleviating local computational pressure and improving system response efficiency. However, both edge nodes and CS are subject to limited computational and communication resources. In particular, when multiple devices concurrently offload tasks to the same node, the computing resources and bandwidth available to each individual task are reduced, resulting in a significant increase in task processing latency. On the other hand, from the task computation model, it can be observed that the task execution delay decreases as more computing resources and transmission bandwidth are allocated, whereas the energy consumption increases accordingly. Therefore, under resource-constrained conditions, achieving a reasonable trade-off between reducing system latency and controlling energy consumption, while designing a jointly optimized TO-RA mechanism, becomes a key challenge in IIoT system design. To address this, we model the TO-RA process as a joint delay–energy optimization problem, aiming to minimize the weighted overall cost incurred during the offloading and execution of all tasks within a given time window, *T*. The optimization problem is formally defined as follows:

(18) $$\begin{array}{cc} \min_{x,f,r}\; & \sum_{t=1}^{T}\sum_{m=1}^{\left|E\right|}\sum_{n=1}^{\left|D\right|}\left(\alpha \cdot T_{n}^{t}+\beta \cdot E_{n}^{t}\right)\\ s.t.\;\; & C1:\;0\leq x_{n,n}^{t}+x_{n,m}^{t}\leq 1,\\ \; & C2:\;\sum_{n\in U_{m}}f_{m,n}^{t}\leq F_{m},\;\sum_{m=1}^{\left|E\right|}f_{cs,n}^{t}\leq F_{cs}\\ \; & C3:\;\sum_{n\in U_{m}}r_{n,m}^{t}\leq R_{m}^{\mathrm{tran}},\;\sum_{m=1}^{\left|E\right|}\leq R_{CS}^{\mathrm{tran}}\\ \; & C4:\;f_{n}^{t}<F_{n} \end{array}$$

The coefficients α and β are non-negative weights satisfying α+β=1, and they are used to balance the trade-off between delay and energy consumption. The optimization problem is subject to constraints, C1–C4. Constraint C1 ensures that the sum of the locally processed data proportion and the offloaded data proportion does not exceed 1. Constraint C2 enforces the computing capacity limits of each ES and the CS. Constraint C3 restricts the bandwidth resource usage to within the transmission limits of each ES and the CS. Constraint C4 ensures that the energy consumption for local computation on mobile devices does not exceed their respective maximum allowable power.

The optimization variables in problem (18) include the TO strategy, computing resource allocation, and uplink rate allocation. These variables are interdependent and involve both discrete and continuous types, making the problem a typical Mixed-Integer Nonlinear Programming (MINLP) problem, which is NP-hard. As a result, it is difficult to obtain the optimal solution to problem (18) within polynomial time, and traditional convex optimization techniques are insufficient for finding the global optimum efficiently. Extensive research has demonstrated the significant potential of DRL in effectively solving MINLP problems. Therefore, in Section 4, we propose a two-stage TO-RA method based on a MADRL algorithm to address this challenge.

## 4. Methods

In this section, we proposes a DPTORA algorithm to address the problem of dynamic priority-aware TO-RA problem in a cloud–edge–end collaborative IIoT environment. The DPTORA algorithm consists of two phases: the first phase solves the TO decision, and the second phase focuses on the allocation of computing and bandwidth resources on the ES after receiving the offloading results.

### 4.1. Stage I: Task Offloading Based on Improved MAPPO

In the cloud–edge–end collaborative architecture, each ES is modeled as an independent agent that continuously observes real-time information from its associated IIoT devices, including task requests, computational capacity, and wireless channel conditions. Based on a dynamic priority-aware mechanism and the current system load, each agent determines the optimal TO strategy. However, due to partial observability of device states, dynamic changes in task priorities, and the uncertainties of network environments, TO decisions cannot rely solely on instantaneous observations. Instead, historical observations must be integrated to make more reasonable decisions. To address the optimization problem (18) in Section 2, we model it as a Partially Observable Markov Decision Process (POMDP)  [43]. The following provides detailed definitions of the state space, observation space, action space, and reward function of the POMDP model.

- **State S:** the global state space $S_{t}$ represents the overall status of the IIoT system at time *t*, encompassing the computing resources, communication resources, and task queue states of all ES:
  (19) $$S_{t}=\left\{S_{t}^{1},S_{t}^{2},\ldots ,S_{t}^{N}\right\}$$
- **Observation O:** The observation space characterizes all the jointly observable information. At the beginning of each time slot *t*, agent *i* obtains its local observation $o_{t}^{i}\in S_{t}$, but it cannot directly access the full global state. The local observation of agent *i* at time *t* is defined as:
  (20) $$o_{t}^{i}=\left\{R_{i}\left(t\right),Q_{i}\left(t\right),F_{i}\left(t\right)\right\}$$
  where $R_{i}\left(t\right)$ denotes the observed task information, including the task size, the required CPU cycles, and the deadline. $Q_{i}\left(t\right)$ indicates the current task queue length and task priority features. $F_{i}\left(t\right)$ reflects the edge server’s available power, computing resources, load level, and connection status with IIoT devices and the cloud.
- **Action A:**A denotes the joint action space of all agents. Based on its local observation and offloading policy, each agent selects the action that maximizes expected reward. The action of agent *i* at time *t* is defined as follows:
  (21) $$a_{t}^{i}=\left\{x_{n,n}^{t},x_{n,m}^{t}\right\}\in A$$
  where the action determines the proportions of the task processed locally and offloaded to the cloud, respectively.
- **Reward R:** The reward is the feedback received from the environment based on the agents’ actions. The reward function is designed to minimize total task delay and energy consumption while encouraging the completion of high-priority tasks:
  (22) $$r_{t}=-\sum_{m=1}^{\left|E\right|}\sum_{n=1}^{\left|D\right|}\left(\omega_{1}G\left(t\right)+\omega_{2}T_{n}^{t}+\omega_{3}E_{n}^{t}\right)$$
  where G(t) evaluates whether a task is completed within its tolerable delay threshold, $\phi_{n}^{t}$. If a task misses the deadline, a penalty proportional to its dynamic priority, $P_{i}^{t,dyn}$, is imposed:
  (23) $$G\left(t\right)=\left\{\begin{array}{cc} 1, & \mathrm{if}\;T_{n}^{t}\leq \phi_{n}^{t}\\ -\theta P_{i}^{t,dyn}, & \mathrm{otherwise} \end{array}\right.$$

#### Improved MAPPO Algorithm

In POMDP problems, due to the large number of tasks and computing nodes, it is difficult for a single agent to handle the high-dimensional state space. Meanwhile, the dynamic changes in task loads and network conditions make it difficult for a single agent to make globally optimal decisions. As one of the most effective MADRL algorithms, MAPPO overcomes the instability issues inherent to multi-agent learning while retaining the high sample efficiency of PPO [44,45]. However, when confronted with dynamically changing task priorities and task-intensive scenarios in IIoT environments, the policy network of MAPPO lacks sufficient capacity to model task priority information, making it difficult to identify and prioritize urgent tasks. This leads to suboptimal decision accuracy and delayed responses under high-pressure conditions. Furthermore, MAPPO’s lack of historical task modeling limits its ability to perceive evolving task trends, thus affecting global adaptability and policy stability.

**Figure 2:**
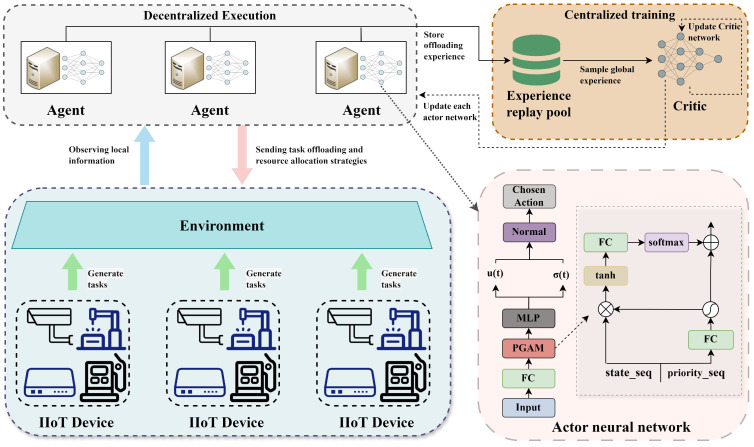
Overall algorithm architecture of the improved MAPPO algorithm.

To address these issues, this study proposes an improved MAPPO-based task offloading algorithm under the CTDE framework, as illustrated in Figure 2. Each ES is treated as an independent agent that interacts with terminal devices and the cloud server to make task offloading decisions. Each agent maintains an independent actor network to make distributed decisions based on local observations and stores experiences in a shared global replay buffer. During training, all agents learn a centralized value function $V_{\phi }\left(S_{t}\right)$ using global state information to update their policies.

In dynamic-priority task-offloading scenarios, the standard MAPPO actor relies solely on the current observation $o^{i}$ to select offloading actions [46], which makes it difficult to capture inter-task priority differences effectively. To address this limitation, we introduce PGAM placed before the original policy network $\pi_{\theta_{i}}$; the overall architecture is shown in Figure 2. PGAM is designed to inject task-priority information directly into feature extraction and to highlight salient tasks via an attention mechanism, thereby enhancing the actor’s perception and decision-making under dynamic-priority conditions. Concretely, PGAM comprises three stages. First, a priority-gating mechanism maps the priority vector to gating signals to modulate state features, amplifying high-priority tasks while suppressing less-relevant ones. Second, an attention-scoring stage assigns weights to the gated feature sequence, enabling the model to focus on more informative historical states. Third, a weighted-aggregation stage produces a context vector that captures the interplay between task priorities and state dependencies. Implementation-wise, PGAM uses standard fully connected layers, a sigmoid-based gating function, and additive attention with softmax normalization, ensuring reproducibility and compatibility with the MAPPO framework while increasing sensitivity to dynamic priorities and improving overall scheduling performance. We next describe the action-generation pipeline in detail.

To provide temporal context, each agent maintains a FIFO buffer that stores the last *T* local observations (T=8). At time step *t*, the buffer contains the observations ${\left\{\left(s_{k}^{i},p_{k}^{i}\right)\right\}}_{k=t-T+1}^{t}$, where $s_{k}^{i}$ and $p_{k}^{i}$ represent the state and priority vectors at time step *k*. These historical observations are processed using the same feature transformation and gating mechanism applied to each time step:

(24) $$x_{k}^{i}=W_{f}s_{k}^{i}+b_{f},\;g_{k}^{i}=\sigma \left(W_{g}p_{k}^{i}+b_{g}\right),\;{\tilde{x}}_{k}^{i}=x_{k}^{i}⊙g_{k}^{i}.$$

The gated feature sequence is then passed through an attention mechanism that computes attention scores over this historical sequence. To ensure that the model focuses only on past observations and not future ones, a causal mask is applied during the attention computation. Specifically, the unnormalized attention score $e_{t,k}^{i}$ for step *k* is computed as follows:

(25) $$e_{t,k}^{i}=w_{a}^{⊤}\tanh\left({\tilde{x}}_{k}^{i}+b_{a}\right),$$

and the normalized attention weight $\alpha_{t,k}^{i}$ for position k∈[t−T+1,t] is computed as follows:

(26) $$\alpha_{t,k}^{i}=\frac{\exp\left(e_{t,k}^{i}+m_{t,k}\right)}{\sum_{\ell =t-T+1}^{t}\exp\left(e_{t,\ell }^{i}+m_{t,\ell }\right)},$$

where $m_{t,k}=0$ for valid historical steps and $m_{t,k}=-\infty$ for future positions (ensuring no future steps are considered). Finally, the context vector is computed by aggregating the weighted feature vectors:

(27) $$c_{t}^{i}=\sum_{k=t-T+1}^{t}\alpha_{t,k}^{i}\cdot {\tilde{x}}_{k}^{i},$$

which is then passed into the policy network to produce an action probability distribution:

(28) $$\pi_{\theta_{i}}\left(a_{t}^{i}|c_{t}^{i}\right)=\mathrm{Softmax}\left(Wc_{t}^{i}+b\right).$$

where *W* and *b* are the weight and bias parameters of the policy network, respectively. The Softmax function ensures that the output forms a valid probability distribution. Finally, the agent samples an action from this distribution:

(29) $$a_{t}^{i}∼\pi_{\theta_{i}}\left(a_{t}^{i}|c_{t}^{i}\right)$$

During the policy update phase, to enhance training stability and prevent drastic policy shifts from adversely affecting task offloading performance, the actor network employs a clipped objective function during optimization:

(30) $$L_{\mathrm{actor}}\left(\theta_{i}\right)=E_{t}\left[\min\left(r_{i}^{t}{\hat{A}}^{t},\;\mathrm{clip}\left(r_{i}^{t},1-\epsilon ,1+\epsilon \right){\hat{A}}^{t}\right)\right]$$

where $r^{t}\left(\theta \right)=\frac{\pi_{\theta }\left(a^{t}|s^{t}\right)}{\pi_{\theta_{\mathrm{old}}}\left(a^{t}|s^{t}\right)}$ is the ratio of the new policy to the old policy, ϵ is the clipping threshold to control the update magnitude, and ${\hat{A}}^{t}$ is the advantage function estimating the quality of the current action relative to the average policy. The advantage function is estimated using the following:

(31) $${\hat{A}}^{t}=\delta_{t}+\left(\gamma \lambda \right)\delta_{t+1}+\cdots +{\left(\gamma \lambda \right)}^{T-t+1}\delta_{T-1}$$

where $\delta_{t}=r_{t}+\gamma V_{\phi }\left(s_{t+1}\right)-V_{\phi }\left(s_{t}\right)$ is the temporal difference (TD) error, and λ is the weight parameter in generalized advantage estimation (GAE) that balances bias and variance.

During centralized training, MAPPO utilizes a centralized Critic network to estimate the global value $V_{\phi }\left(S_{t}\right)$, thereby improving the accuracy of system-wide value estimation:

(32) $$V_{\phi }\left(S_{t}\right)=f_{\mathrm{Critic}}\left(S_{t}\right)$$

where the critic network is parameterized by ϕ and is updated by minimizing the TD error, with the loss function defined as follows:

(33) $$L_{\mathrm{critic}}\left(\phi \right)=\frac{1}{N}\sum_{i=1}^{N}E_{t}\left[{\left(r_{t}+\gamma V_{\phi }\left(S_{t+1}\right)-V_{\phi }\left(S_{t}\right)\right)}^{2}\right]$$

where *N* is the number of agents, $r_{t}$ is the immediate reward at time step *t*, and γ is the discount factor representing the weight of future rewards.

Finally, both the actor and critic networks are updated through gradient descent as follows:

(34) $$\begin{array}{cc} \theta_{i} & \leftarrow \theta_{i}-\eta_{\pi }\nabla_{\theta_{i}}L_{\mathrm{actor}}\left(\theta_{i}\right) \end{array}$$

(35) $$\begin{array}{cc} \phi & \leftarrow \phi -\eta_{v}\nabla_{\phi }L_{\mathrm{critic}}\left(\phi \right) \end{array}$$

where $\eta_{\pi }$ and $\eta_{v}$ are the learning rates for the policy and value networks, respectively. The update procedure of the proposed algorithm is illustrated in Algorithm 2.
**Algorithm 2** Improved MAPPO for task offloading  1: **Input:** Edge computing environment parameters, Agent set A, Local observations $o_{t}^{i}$, Policy network parameters $\theta^{i}$, Centralized Critic network parameters ϕ, Experience replay buffer D, Learning rates $\eta_{\pi }$, $\eta_{v}$.
  2: **Output:** Task offloading policy $\pi_{\theta^{i}}$
  3: Initialize policy networks $\pi_{\theta^{i}}$ for each agent
  4: Initialize global critic network $V_{\phi }$
  5: Initialize experience buffer D
  6: **for** episode = 1 **to** *E* **do**
  7:      Initialize global state $S_{0}$
  8:      **for** t=1 **to** *T* **do**
  9:             **for** each agent i∈A **do**
10:                   Observe local state $o_{t}^{i}$
11:                   Calculate context vector $c_{t}^{i}$ via PGAM
12:                   Sample action $a_{t}^{i}∼\pi_{\theta^{i}}\left(\cdot |c_{t}^{i}\right)$
13:                   Execute $a_{t}^{i}$, receive reward $r_{t}^{i}$, observe $o_{t+1}^{i}$
14:                   Compute global state:
        $S_{t}=f_{\mathrm{agg}}\left({\left\{o_{t}^{j}\right\}}_{j=1}^{\left|A\right|}\right)$
15:                   Store $\left(S_{t},\left\{o_{t}^{i},h_{t}^{i},a_{t}^{i},r_{t}^{i}\right\},S_{t+1}\right)$ in buffer D
16:             **end for**
17:             **for** mini-batch k=1 **to** *K* **do**
18:                   Sample mini-batch from D
19:                   Compute value: $V_{\phi }\left(S_{t}\right)=f_{\mathrm{Critic}}\left(S_{t}\right)$
20:                   Compute target:        ${\hat{V}}_{\phi }\left(S_{t}\right)=r_{t}+\gamma V_{\phi }\left(S_{t+1}\right)$
21:                   Compute advantage:        $A_{t}={\hat{V}}_{\phi }\left(S_{t}\right)-V_{\phi }\left(S_{t}\right)$
22:                   Update critic:        $\phi \leftarrow \phi -\eta_{v}\nabla_{\phi }L_{\mathrm{critic}}\left(\phi \right)$
23:                   Update actor:        $\theta^{i}\leftarrow \theta^{i}-\eta_{\pi }\nabla_{\theta^{i}}L_{\mathrm{actor}}\left(\theta^{i}\right)$
24:             **end for**
25:      **end for**
26: **end for**


### 4.2. Stage II: Resource Allocation Phase

After completing TO in Stage I, each ES and CS must formulate a resource allocation strategy based on the offloading actions, the remaining computational resources, and the available bandwidth. This strategy is used to handle multiple IIoT tasks received at time step *t*, aiming to minimize the total transmission and computation delays while improving system resource utilization.

As modeled in Section 3, the total delay at each ES *m* at time *t* is as follows:

(36) $$T_{E_{m}}^{t}=\sum_{n=1}^{|U_{m}|}\left[\left(1-x_{n,n}^{t}\right)\cdot x_{n,m}^{t}\left(\frac{d_{n}^{t}}{r_{n,m}^{t}}+\frac{c_{n}^{t}}{f_{m,n}^{t}}\right)\right]$$

In this phase, the offloading ratio, task size, and computation cycles are all treated as known constants. Let $\alpha_{n}=\left(1-x_{n,n}^{t}\right)\cdot x_{n,m}^{t}$. Since total delay is inversely proportional to bandwidth and computation resource allocations, the resource allocation problem for ES is formulated as follows:

(37) $$\begin{array}{cc} \min_{r_{n,m}^{t},f_{m,n}^{t}}\; & f\left(r_{n,m}^{t},f_{m,n}^{t}\right)=\sum_{n=1}^{|U_{m}|}\alpha_{n}\left(\frac{d_{n}^{t}}{r_{n,m}^{t}}+\frac{c_{n}^{t}}{f_{m,n}^{t}}\right)\\ s.t.\; & \sum_{n=1}^{|U_{m}|}r_{n,m}^{t}=R_{m}^{\mathrm{tran}},\;r_{n,m}^{t}>0\\ \; & \sum_{n=1}^{|U_{m}|}f_{n,m}^{t}=F_{m},\;f_{n,m}^{t}>0 \end{array}$$

The objective function terms $\frac{d_{n}^{t}}{r_{n,m}^{t}}$ and $\frac{c_{n}^{t}}{f_{m,n}^{t}}$ are convex with respect to $r_{n,m}^{t}$ and $f_{m,n}^{t}$, respectively, as their second derivatives are greater than zero. All constraints are linear, forming a convex feasible set. Hence, the optimization problem is a convex minimization problem. We can exploit the duality of the objective using KKT conditions. By constructing the Lagrangian and introducing Lagrange multipliers, the transmission and computation resource constraints can be decoupled. This leads to the derivation of a closed-form global optimal solution for bandwidth and computational resource allocation [47]. The specific process of resource allocation is provided in Algorithm 3:
**Algorithm 3** Resource allocation algorithm for edge server
  1: **Input:** Edge server set *M*; task proportion coefficients $\alpha_{n}$; task demand $d_{n}^{t}$; computation demand $c_{n}^{t}$; bandwidth $B_{m}$; CPU capacity $F_{m}$; convergence threshold ϵ
  2: **Output:** Optimal bandwidth and CPU allocation $r_{n,m}^{t*},f_{m,n}^{t*}$
  3: **for** each edge server m∈M **do**
  4:       Initialize Lagrange multipliers $\lambda_{m}^{\left(0\right)}$, $\mu_{m}^{\left(0\right)}$ with arbitrary positive values
  5:       **repeat**
  6:             Update Lagrange multipliers $\lambda_{m}$, $\mu_{m}$
  7:             Check convergence conditions:             $|\lambda_{m}-\lambda_{m}^{\left(\mathrm{prev}\right)}\left|<\epsilon \;\mathrm{and}\;\right|\mu_{m}-\mu_{m}^{\left(\mathrm{prev}\right)}|<\epsilon$
  8:             **if** converged **then**
  9:                   **break**
10:             **else**
11:                   Set $\lambda_{m}^{\left(\mathrm{prev}\right)}\leftarrow \lambda_{m}$, $\mu_{m}^{\left(\mathrm{prev}\right)}\leftarrow \mu_{m}$
12:        **end if**
13:       **until** convergence
14:       Compute optimal allocation: $r_{n,m}^{t*},f_{m,n}^{t*}$
15: **end for**


First, we construct the Lagrangian:

(38) $$\begin{array}{cc} L(r_{n,m}^{t},f_{m,n}^{t},\lambda ,\mu )= & \sum_{n=1}^{|U_{m}|}\alpha_{n}\left(\frac{d_{n}^{t}}{r_{n,m}^{t}}+\frac{c_{n}^{t}}{f_{m,n}^{t}}\right)\\ \; & +\lambda \left(\sum_{n=1}^{|U_{m}|}r_{n,m}^{t}-R_{m}^{\mathrm{tran}}\right)\\ \; & +\mu \left(\sum_{n=1}^{|U_{m}|}f_{n,m}^{t}-F_{m}\right) \end{array}$$

where λ and μ are the Lagrange multipliers. Taking the partial derivatives and setting them to zero, we obtain the following:

(39) $$\frac{\partial L}{\partial r_{n,m}^{t}}=-\frac{\alpha_{n}d_{n}^{t}}{{\left(r_{n,m}^{t}\right)}^{2}}+\lambda =0\;\Rightarrow \;r_{n,m}^{t}=\sqrt{\frac{\alpha_{n}d_{n}^{t}}{\lambda }}$$

(40) $$\frac{\partial L}{\partial f_{m,n}^{t}}=-\frac{\alpha_{n}c_{n}^{t}}{{\left(f_{m,n}^{t}\right)}^{2}}+\mu =0\;\Rightarrow \;f_{m,n}^{t}=\sqrt{\frac{\alpha_{n}c_{n}^{t}}{\mu }}$$

Substituting the above into the resource constraints, we solve for the multipliers:

(41) $$\sum_{n=1}^{|U_{m}|}\sqrt{\frac{\alpha_{n}d_{n}^{t}}{\lambda }}=R_{m}^{\mathrm{tran}}\;\Rightarrow \;\lambda ={\left(\frac{\sum_{n=1}^{|U_{m}|}\sqrt{\alpha_{n}d_{n}^{t}}}{R_{m}^{\mathrm{tran}}}\right)}^{2}$$

(42) $$\sum_{n=1}^{|U_{m}|}\sqrt{\frac{\alpha_{n}c_{n}^{t}}{\mu }}=F_{m}\;\Rightarrow \;\mu ={\left(\frac{\sum_{n=1}^{|U_{m}|}\sqrt{\alpha_{n}c_{n}^{t}}}{F_{m}}\right)}^{2}$$

Finally, the optimal resource allocation strategies are as follows:

(43) $$r_{n,m}^{t*}=\sqrt{\frac{\alpha_{n}d_{n}^{t}}{\lambda }},\;f_{m,n}^{t*}=\sqrt{\frac{\alpha_{n}c_{n}^{t}}{\mu }}$$

### 4.3. Computational Complexity Analysis of DPTORA

In this subsection, we analyze the computational complexity of the proposed DPTORA algorithm from both time and space complexity perspectives. The overall time complexity of DPTORA integrates the complexities of the MAPPO-based TO decision-making and the KKT-based resource allocation optimization.

During the TO phase, each ES acts as an independent agent making decisions through a deep neural network. The inference complexity is primarily determined by the forward propagation of the policy network, with a time complexity of O(MF), where *M* denotes the number of ESs and *F* denotes the number of floating-point operations required for a single forward pass of the neural network. In the resource allocation phase, the optimal bandwidth and computation resources are calculated using an iterative method based on the KKT conditions. For each ES, if it needs to handle at most *N* tasks, and the iterative process requires *I* iterations to converge, then the complexity for this phase is O(MIN). Therefore, the worst-case time complexity of the DPTORA algorithm for each time slot is O(MF+MIN).

The space complexity of DPTORA mainly arises from the deep learning model parameters and the data structures used for KKT optimization. Each ES maintains a policy network and a value network, with a total parameter size of O(M|θ|), where |θ| represents the number of parameters in each network. Additionally, during training, the experience replay buffer stores observations and actions of all agents, requiring memory of size O(MB(d+a)), where *B* is the batch size, *d* is the observation dimension, and *a* is the action space dimension. The KKT optimization process also requires storage for Lagrange multipliers, with a space complexity of O(MN). Hence, the overall space complexity of the algorithm can be summarized as follows: O(M|θ|+MB(d+a)+MN)

## 5. Results

In this section, we conduct extensive experiments to validate the performance of the proposed DPTORA algorithm. To demonstrate its feasibility, we compare DPTORA with several baselines and the most advanced MADRL algorithms, as follows:

1. Local-only scheduling baseline algorithm: This fully decentralized baseline executes all computation tasks locally on each IIoT device without any offloading. It serves as a lower bound to reflect performance in the absence of collaborative scheduling.
2. Random scheduling baseline algorithm [48]: This baseline randomly assigns each generated task to the local device, an edge server, or the cloud. As a non-intelligent comparator, it highlights the advantages of our method in terms of rational scheduling and performance optimization.
3. MADDPG algorithm [30,49]: MADDPG is tailored for cooperative multi-agent tasks and is suitable for environments with coupled agent interactions. In task-offloading settings, MADDPG can learn coordinated policies across heterogeneous devices and resource constraints, thereby improving overall system performance; it thus serves to verify DPTORA’s advantages under complex cooperative scheduling.
4. MAPPO algorithm [22,44,50]: MAPPO is an on-policy deep reinforcement learning algorithm for multi-agent scenarios, following a centralized-training–decentralized-execution actor–critic paradigm. It is known for stable learning and convergence in complex environments and can optimize offloading policies under dynamic network conditions and concurrent workloads, making it a strong DRL baseline for task scheduling and resource allocation.

### 5.1. Experimental Setup

The simulation platform was implemented on a workstation equipped with an Intel Core i9-13905H (2.40 GHz) and 32 GB RAM, using Python 3.9.17 and PyTorch 2.0.1. The experimental scenario initially comprises 10 IIoT devices, 3 ES, and 1 cloud server. IIoT devices connect via Wi-Fi and are evenly assigned to distinct edge servers, while edge servers connect to the cloud over fiber broadband. In the simulator, state observability and action execution are exposed directly through Python APIs, requiring no additional communication protocols. We assume that, in each time slot, task arrivals at IIoT devices follow a normal distribution with a mean of 2. In the reward design, the weighting factors for deadline violations, total latency, and total energy consumption are set to 0.3, 0.5, and 0.2, respectively, to balance multiple objectives. Detailed simulation parameters are summarized in Table 3.

In the experiments, ES hosts an independent actor, while a centralized critic is shared across agents. The actor incorporates PGAM at the input, followed by two fully connected layers with 128 hidden units each and ReLU activations. The centralized critic consists of three fully connected layers with 256, 512, and 128 hidden units, respectively, using ReLU, to estimate the global value function. Both networks are trained with Adam (learning rate $3\times 10^{-4}$), discount factor γ=0.99, generalized advantage estimation (GAE) parameter λ=0.95, PPO clip ratio 0.4, soft-update coefficient τ=0.01, and entropy coefficient 0.01; each update comprises 15 training epochs. Training spans 600 episodes, with each consisting of 300 time steps.

### 5.2. Convergence Performance Evaluation

#### 5.2.1. Performance Comparison of Convergence

This subsection evaluates the learning efficiency and policy quality of each algorithm during training by comparing the convergence behavior of DPTORA against several baselines across two experimental settings, using the average cumulative reward as the primary evaluation metric.

**Figure 3:**
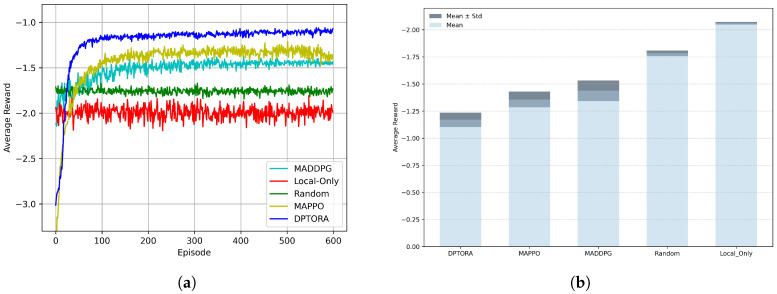
(**a**) Average reward comparison under different algorithm. (**b**) Mean and variance of the average return across repeated runs with different random seeds.

To evaluate the learning efficiency and policy quality of each algorithm during training, this section compares the convergence performance of DPTORA with five baseline strategies based on the average cumulative reward. Figure 3a illustrates the reward trends of different algorithms over training episodes, where the horizontal axis denotes the number of iterations and the vertical axis represents the average reward per episode. As shown in the figure, DPTORA demonstrates the best convergence performance, reaching a stable state around the 60th to 70th iteration, with the average reward stabilizing near −1.15 and exhibiting lower variance than other algorithms. This indicates that DPTORA can learn high-quality offloading policies more rapidly, achieving efficient resource scheduling and optimized task response times.

In contrast, MAPPO converges around the 100th iteration with a final average reward of approximately −1.35. We attribute its performance disadvantage mainly to its insufficient modeling capability of partially observable environments. In dynamic IIoT scenarios, agents have access only to partial local information, while the native MAPPO architecture lacks a mechanism to model historical information, making it difficult to accurately estimate hidden states. This results in unstable policy learning and increased bias in value estimation. DPTORA incorporates the PGAM into the actor network to effectively integrate historical state sequences via an attention mechanism. By leveraging a gating mechanism, it enhances the perception of task priority features, thereby guiding the policy to prioritize critical tasks. This design accelerates policy convergence and improves overall performance. Although MADDPG also employs a centralized training and decentralized execution framework, it performs poorly in this task scenario, with a final average reward around −1.43. This is primarily because MADDPG’s actor networks rely on instantaneous observations without historical state modeling, making it challenging to adapt to complex tasks and network dynamics. Moreover, its critic network must process the joint actions of all agents, causing dimensionality to explode as the number of agents increases, which destabilizes gradient estimation and impairs training convergence.

The traditional Local-Only Scheduling and Random Scheduling algorithms represent two extreme cases of fully local computation and full offloading, respectively, and fail to account for the dynamic variation of tasks and resource states. The former depends entirely on terminal devices for task execution, limited by computing capacity, leading to high latency and energy consumption; the latter blindly relies on edge computing, easily causing node congestion and queuing delays. Experimental results show that both exhibit oscillatory average rewards within fixed intervals without clear convergence trends, reflecting their poor adaptability and robustness in dynamic IIoT environments.

Furthermore, we performed five independent runs of DPTORA under different random seeds (42, 321, 899, 1066, 3407). Figure 3b shows the individual convergence trajectories and the corresponding mean curve, with a semi-transparent shaded band around the mean denoting ±1 standard deviation. The five single-run curves exhibit highly consistent trends and converge to similar steady values, indicating that random initialization has a limited impact on the training outcomes. Meanwhile, the narrow shaded band around the mean indicates small performance variance across seeds, evidencing strong robustness and reproducibility. Overall, DPTORA maintains fast convergence and high asymptotic performance in dynamic IIoT scenarios and delivers stable, consistent results across independent runs, further substantiating the reliability of the proposed method.

Overall, DPTORA significantly enhances the estimation of hidden states in partially observable environments through structural improvements in state modeling and policy stability. Additionally, in the second phase of the algorithm, a globally optimal bandwidth and computational resource allocation strategy is derived based on the Lagrangian dual method, which effectively reduces the variance of policy gradients and improves the stability and convergence speed of training. Compared with existing methods, DPTORA demonstrates superior performance and stronger adaptability in complex and dynamic environments.

#### 5.2.2. Ablation Experiment

**Figure 4:**
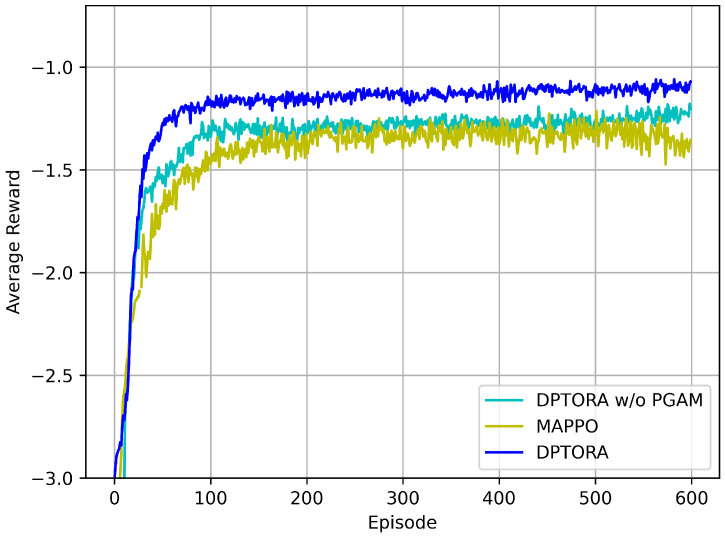
Comparison of average reward curves among DPTORA, DPTORA w/o PGAM, and MAPPO algorithms.

This section conducts representative ablation studies to evaluate the effectiveness of the proposed PGAM module in capturing dynamic priorities and improving scheduling performance. Under dynamic-priority settings, we compare the average reward of DPTORA, DPTORA without PGAM (DPTORA w/o PGAM), and MAPPO. As shown in Figure 4, the performance curves of DPTORA w/o PGAM and MAPPO nearly overlap and are substantially lower than that of the full DPTORA. These results indicate that relying solely on MAPPO for task offloading is insufficient to achieve performance comparable to DPTORA. Further analysis shows that with PGAM, the model employs an attention mechanism to weight and integrate historical state sequences and uses a gating structure to amplify priority features, enabling the policy to identify critical tasks more accurately and prioritize their scheduling in dynamic-priority environments, thereby accelerating convergence and improving the final average reward. Therefore, PGAM is a key component underpinning DPTORA’s performance advantage for task offloading with dynamic priorities.

### 5.3. Scalability and Load Adaptability Evaluation

#### 5.3.1. Convergence Analysis Under Different Numbers of Devices

**Figure 5:**
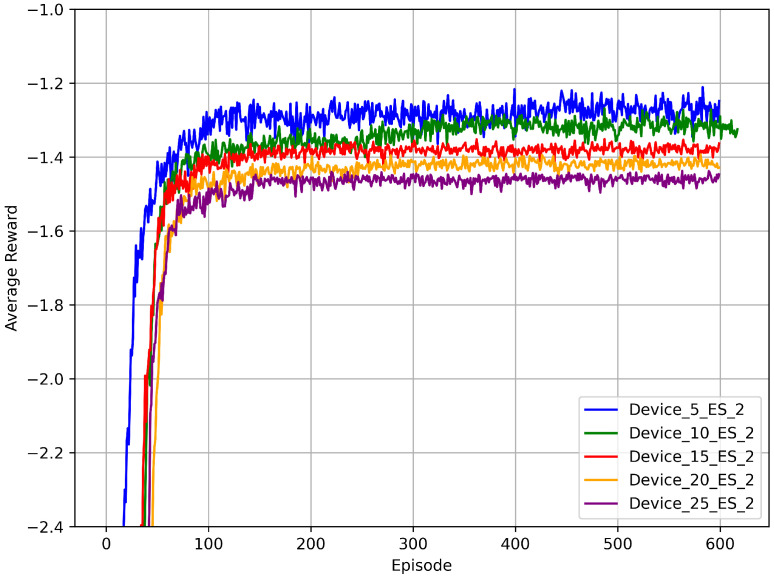
Comparison of average rewards across different device quantities.

To evaluate the learning stability and policy generalization ability of DPTORA under an increasing IIoT device scale, we fixed the number of edge servers at 2 and conducted experiments with varying device counts. Figure 5 presents the trend of average rewards over training episodes for different device scales. Although the task density significantly increases with more devices, DPTORA still exhibits good convergence performance across all scales. When the device count reaches 25, the algorithm stabilizes around 100 training episodes, demonstrating strong scalability. Moreover, as the number of devices increases and the system load grows, the final average reward slightly declines; however, the overall trend remains smooth, indicating strong adaptability and robustness under high-load conditions.

#### 5.3.2. Impact of Device Scale on System Performance

**Figure 6:**
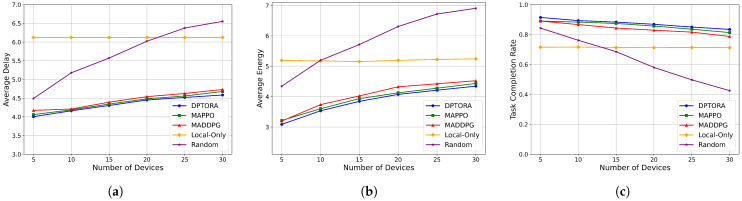
(**a**) Comparison of the average delay of different numbers of devices. (**b**) Comparison of the average energy of different numbers of devices. (**c**) Comparison of the task completion rate of different numbers of devices.

To further verify the system scheduling capability of DPTORA under different task and equipment scales, we evaluated five algorithms based on three performance metrics: average latency, average energy consumption, and task completion rate. The evaluation was conducted while progressively increasing the number of devices, with the number of edge servers held constant at 2. The results are shown in Figure 6.

Figure 6a shows that, except for Local-only Scheduling, average latency increases significantly for the other four algorithms as the device count grows. This is mainly due to resource sharing among more tasks, which prolongs computation and data transmission times. Local-only scheduling remains relatively unaffected due to its complete reliance on local execution, though its baseline latency is higher. DPTORA consistently achieves the lowest latency and smallest growth rate, demonstrating its efficiency in joint TO-RA. Figure 6b shows that energy consumption trends are consistent with latency. Most algorithms show increased energy consumption due to either local execution demands or the need for higher transmission power. DPTORA maintains a clear advantage in energy efficiency, with a lower growth rate compared to MAPPO and MADDPG, indicating effective load balancing under high-density conditions. In Figure 6c, random scheduling shows a sharp decline in task completion rate as device number increases, due to a lack of coordination. MADDPG, MAPPO, and DPTORA maintain rates above 0.9. Among them, DPTORA achieves the highest completion rates due to dynamic joint modeling of offloading strategies and resource allocation, improving scheduling success rates. In summary, DPTORA consistently maintains significant advantages in key performance metrics such as task response delay, energy consumption, and task completion rate, even as the number of devices increases. This demonstrates its excellent scalability and scheduling stability.

#### 5.3.3. Impact of Edge Server Quantity on System Performance

**Figure 7:**
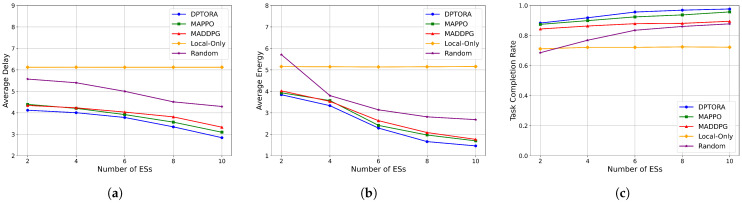
(**a**) Comparison of the average delay of different numbers of ESs. (**b**) Comparison of the average energy of different numbers of ESs. (**c**) Comparison of the task completion rate of different numbers of ESs.

To evaluate the effect of edge resource scaling, we fixed the number of terminal devices at 15 and gradually increased the number of edge servers. The performance of five algorithms was compared on four metrics: average reward, latency, energy consumption, and task completion rate. The results are presented in Figure 7.

As shown in Figure 7a, local-only scheduling remains constant in latency due to its non-reliance on edge resources. Other algorithms experience decreasing latency with more edge servers due to enhanced computing and bandwidth capacity. DPTORA achieves the largest latency reduction from 2 to 10 edge servers, showing its strong ability to utilize additional resources effectively. Figure 7b indicates that energy consumption decreases with edge resource expansion, consistent with latency trends. Closer edge nodes reduce transmission power requirements and local computation burdens. DPTORA exhibits the lowest energy consumption, indicating effective task-resource balancing. In Figure 7c, DPTORA’s task completion rate rises rapidly with more edge servers, surpassing 95% at six servers and stabilizing afterward. In contrast, MADDPG and traditional methods improve slowly, limited by weaker adaptability or convergence. Based on the above analysis, DPTORA demonstrates excellent performance responsiveness and resource utilization efficiency as the number of edge servers increases. In particular, it exhibits a superior scheduling capability and marginal resource gains under task-intensive conditions, confirming its strong adaptability to resource scaling in practical deployments.

### 5.4. Performance Comparison in Dynamic Priority Task Scenarios

**Figure 8:**
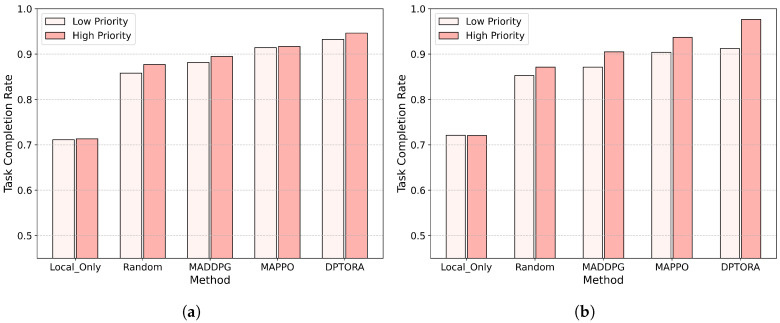
(**a**) Comparison of task completion rates in static priority task sets. (**b**) Comparison of task completion rates in dynamic priority task sets.

This section investigates the influence of dynamic task priority mechanisms on the performance of various algorithms. To this end, we design two types of task sets: static priority and dynamic priority. In the static priority task set, task priorities remain unchanged after generation, while in the dynamic priority task set, task urgencies are adjusted during execution based on the rules of the dynamic priority queue model described in Section 3. We evaluate and compare the completion performance of high- and low-priority tasks for each algorithm. The experimental results are presented in Figure 8.

As shown in Figure 8a, DPTORA achieves the highest completion rate for high-priority tasks, reaching nearly 95%. This is significantly better than the other algorithms, particularly random scheduling and local-only strategies. These results demonstrate that, under constrained edge resource environments, DPTORA can effectively prioritize the execution of critical tasks through its dynamic resource scheduling and adaptive offloading strategy. For low-priority tasks, although the differences among algorithms are smaller, DPTORA still maintains a slight advantage. This indicates that DPTORA not only emphasizes the completion of critical tasks but also avoids severely degrading the service quality for less urgent tasks, achieving a balanced scheduling effect.

Figure 8b illustrates the algorithm performance under dynamic priority task conditions. DPTORA again outperforms all other methods, maintaining a high-priority task completion rate exceeding 97%. This result highlights the effectiveness of the PGAM module in the task offloading phase, where the gated structure enables explicit modeling of task priority. Consequently, the policy can more accurately focus on urgent tasks, enhancing its responsiveness and increasing the likelihood of high-priority tasks being successfully completed. In contrast, the completion rates of MAPPO and MADDPG show little change compared to the static case, indicating that their policy architectures lack sensitivity to evolving task urgency. Although DPTORA experiences a slight decrease in the completion rate for low-priority tasks, the overall performance remains within acceptable bounds. The combined results of both sets of experiments demonstrate that DPTORA not only possesses the capability of hierarchical scheduling based on task importance in static scenarios but also exhibits superior policy adaptability and scheduling responsiveness in dynamic priority-changing environments.

## 6. Conclusions

In this paper, we investigate the problem of task offloading and resource allocation in IIoT environments characterized by dynamic task priorities. To reduce latency and energy consumption while improving task completion rates, we propose the DPTORA algorithm. During the task offloading phase, DPTORA employs an enhanced MAPPO algorithm, where a PGAM is integrated to explicitly modulate the attention weight distribution. This allows the policy network to focus on priority-aware features, thereby significantly improving decision accuracy in dynamic environments. In the resource allocation phase, DPTORA constructs an optimization model with QoS constraints and derives globally optimal strategies for computing and bandwidth resource allocation using the Lagrangian dual method and KKT conditions, achieving a synergistic improvement in both task processing efficiency and resource utilization. Simulation results demonstrate that DPTORA significantly outperforms benchmark reinforcement learning algorithms such as MAPPO and MADDPG, as well as traditional scheduling strategies, in terms of system convergence speed, average delay, energy consumption, and task completion rate. In complex scenarios with dynamically changing task priorities, DPTORA exhibits superior adaptability and differentiated scheduling capabilities, effectively prioritizing the completion of critical tasks. However, this study adopts several modeling simplifications—for example, the transmission rate between edge servers and the cloud is treated as constant, and network congestion and link fluctuations are not modeled. These assumptions limit the applicability of the model to real-world industrial settings. Future work will relax these assumptions by incorporating more realistic network conditions and multi-level resource constraints, and it will explore cross-domain collaborative optimization to facilitate the deployment of the proposed framework in large-scale IIoT systems.

## Figures and Tables

**Table 1:** Summary of related works.

Work	Year	Optimization Target			Approach	Objective
		Energy	Delay	Priority		
[23]	2019	✓	✓	×	Heuristic Optimization	Minimize system energy and task execution latency
[24]	2019	✓	✓	×	Graph-Based Multi-Hop Offloading Algorithm	Minimize overall task latency and edge energy consumption across IoT
[25]	2024	✓	×	×	Game-theoretic model	Optimize task allocation and resource utilization to minimize energy
[26]	2023	✓	×	×	Non-cooperative Game Theory	Maximize profit defined by minimizing energy and transmission-related costs
[27]	2022	✓	✓	×	Lyapunov Optimization	Minimize energy and task delay via online UAV trajectory and offloading control
[28]	2023	✓	✓	×	Online Reinforcement Learning	Minimize energy and task delay via online UAV trajectory and offloading control
[29]	2023	✓	✓	×	Proximal Policy Optimization	Minimize overall cost of multi-task offloading
[30]	2024	✓	✓	×	Multi-Agent Deep Deterministic Policy Gradient	Minimize long-term average delay and economic cost under QoS constraints
[31]	2023	✓	✓	×	Cooperative Multi-Agent Deep Reinforcement Learning	Optimize offloading efficiency under topology and resource constraints
[35]	2025	✓	×	✓	Deep Reinforcement Learning	Improve completion rate for high-priority tasks
[32]	2023	✓	✓	×	Graph Attention Multi-Agent Reinforcement Learning	Maximize QoE-based system utility under storage and radio resource constraints
[33]	2024	×	✓	✓	Double Dueling Deep Q-Network	Minimize overall task latency
[34]	2023	×	✓	✓	Priority-Aware Deep Deterministic Policy Gradient	Maximize average system utility under dynamic QoS-aware task offloading
Our work	2025	✓	✓	✓	Improved Multi-Agent Proximal Policy Optimization	Jointly optimize task offloading, resource allocation, and priority adaptation to enhance system efficiency

**Table 2:** Notations used in this paper.

Notation	Description
*N*	Number of IIoT devices
*M*	Number of ES
$d_{n}^{t}$	Data size of task $R_{n}^{t}$
$c_{n}^{t}$	CPU cycles for computing task $R_{n}^{t}$
$\phi_{n}^{t}$	Maximum latency of task $R_{n}^{t}$
$x_{n,n}^{t}$	Proportion of task computed locally
$x_{n,m}^{t}$	Proportion of task offloaded to ES
$h_{n,m}^{t}$	Channel gain between IIoT *n* and ES *m*
$P_{n,m}^{t}$	Transmission power at time *t*
$B_{n,m}^{t}$	Bandwidth between device *n* and ES *m*
${\mathrm{SNR}}_{n,m}^{t}$	SNR between device *n* and ES *m*
$R_{m}^{\mathrm{tran}}$	Total bandwidth resources of ES *m*
$R_{\mathrm{CS}}^{\mathrm{tran}}$	Total bandwidth resources of CS
$F_{n}$	CPU frequency of device *n*
$F_{m}$	CPU frequency of ES *m*
$F_{\mathrm{cs}}$	CPU frequency of CS
$E_{n}^{t}$	Total energy consumption of device *n*
$T_{\mathrm{Cloud},n,m}^{t}$	Task latency on the cloud
$T_{n}^{t}$	Total task completion delay

**Table 3:** Simulation parameters.

Notation	Description	Value
$d_{n}$	Data size of task $R_{n}$	150–300 MB
$c_{n}$	CPU cycles required by task $R_{n}$	20–50 Gcycles
$B_{\mathrm{WiFi}}$	WiFi bandwidth	30–50 MHz
$B_{\mathrm{FO}}$	Fiber optic bandwidth	200–300 MHz
$P_{n}$	Transmission power of IIoT device *n*	0.5 W
$\kappa_{n}$	Hardware-related constant for IIoT device *n*	$10^{-28}$
$f_{n}$	CPU frequency of IIoT device *n*	10–30 Gcycles/s
$f_{m}$	CPU frequency of edge server *m*	60–80 Gcycles/s
$f_{cs}$	CPU frequency of cloud server	200 Gcycles/s
$h_{n,m}$	Channel gain between device *n* and server *m*	$2\times 10^{-9}$–$1.8\times 10^{-6}$
$N_{0}$	Noise power	$1.5\times 10^{-10}$ W

## Data Availability

The datasets generated and analyzed during the current study contain sensitive information related to industrial production processes and device operations, which involve potential corporate privacy and security concerns. Therefore, the data cannot be made publicly available. However, the data can be obtained from the corresponding author upon reasonable request, subject to a confidentiality agreement to ensure that the information is not misused.
